# Structural characterization of a polysaccharide from Qi-Gui herb pair and its anti-tumor activity in colon cancer cells 

**DOI:** 10.3389/fphar.2025.1557151

**Published:** 2025-03-24

**Authors:** Wen-Juan Liu, Ye-Zi Ma, Jia-Xin Li, Bei-Sheng Fan, Xiao-Qiang Li, Wei Cao, Yu-Ping Tang

**Affiliations:** ^1^ Key Laboratory of Shaanxi Administration of Traditional Chinese Medicine for TCM Compatibility, Shaanxi University of Chinese Medicine, Xianyang, China; ^2^ Department of Pharmacology and Key Laboratory of Gastrointestinal Pharmacology of Chinese Materia Medica of the State Administration of Traditional Chinese Medicine, School of Pharmacy, Air Force Medical University, Xi’an, China; ^3^ Shaanxi Key Laboratory of Natural Products and Chemical Biology, School of Chemistry and Pharmacy, Northwest A&F University, Yangling, China

**Keywords:** polysaccharide, Qi-Gui herb pair, structural characteristics, anti-tumor, cell cycle, apoptosis

## Abstract

*Astragalus membranaceus* (Fisch.) Bunge and *Angelica sinensis* (Oliv.) Diels forms a classic herb pair (Qi-Gui her pair) in Chinese medicine, which was commonly used for treating menstrual anemia and microvascular ischemic diseases. While polysaccharides are known to be key bioactive components of the Qi-Gui herb pair, their structural characteristics and pharmacological activities remain underexplored. In this research, a homogeneous polysaccharide with a molecular weight of 18.1 kDa was isolated, and its structure was analyzed via high pressure size exclusion chromatography, high performance liquid chromatography, gas chromatography mass spectrometry, and nuclear magnetic resonance spectroscopy. The structural analysis revealed that AAPS-1a was composed of α-T-Glc*p* (5.9%), β-1,3-Gal*p* (3.9%), α-1,4-Man*p* (3.6%), α-1,4-Gal*p* (2.1%), α-1,4-Glc*p* (2.8%), and α-1,6-Glc*p* (81.7%). Furthermore, NMR analysis revealed that AAPS-1a consists of a repeat unit: α-T-Glc*p*-(1→4)-α-Gal*p*-(1→4)-α-Man*p*-(1→4)-α-Glc*p*-(1→[6)-α-Glc*p*-(1]_n_→3)-β-Gal*p*-(1→. *In vitro* studies showed that AAPS-1a could significantly inhibit the proliferation of HCT116 cells, and induces G1 arrest and G2/M arrest, as well as apoptosis of HCT116 cells. This study presents the inaugural report establishing a connection between the structural characteristics of Qi-Gui herbal polysaccharides and their anti-colon cancer activity, demonstrating that AAPS-1a holds promise as a therapeutic agent for the treatment of colon cancer.

## 1 Introduction

Colon cancer is a prevalent malignant neoplasm of the digestive tract and is one of the top three malignancies worldwide (Sung et al., 2021). Surgical intervention constitutes the primary treatment modality for colon cancer, with a reported 5-year survival rate of approximately 50% post-surgery. However, due to the indistinct primary tumor site and the subtlety of initial symptoms, approximately 83% of patients are diagnosed at advanced stages (Sung et al., 2021). Moreover, the therapeutic efficacy of combining surgery with chemoradiotherapy remains suboptimal, resulting in a significant incidence of recurrence and metastasis among patients. Immunotherapy shows promise in cancer treatment by boosting immune responses with engineered T-cell receptors like ImmTACs, using safety switches for modified T cells, and applying targeted therapies like anti-PD1/PDL1 and CDK4/6 inhibitors (Zhao et al., 2021; Jin et al., 2019). It also involves researching prognostic genes in the tumor microenvironment, such as CASKIN1, EMR3, and GBP5, studying epigenetics in gastric cancer, and evaluating LCK as a melanoma biomarker (Xiang et al., 2021; Yang et al., 2022; Wang et al., 2022). In addition, nanozyme-mediated catalytic therapy, enzyme-driven nanomotors, and metal-based nanomaterials hold significant potential to enhance therapeutic efficiency by boosting ROS production, targeting tumors, and addressing enzyme fragility (Yue et al., 2024; Tang et al., 2024; Lei et al., 2023). Progress in NIR-II imaging for detecting tumor heterogeneity and the contribution of metal-based materials in enhancing radiotherapy, catalytic therapy, and immunotherapy are vital, providing new perspectives for non-invasive, effective cancer treatments (Xin et al., 2023; Zhou et al., 2024; Huang et al., 2024). However, due to immune-related side effects and individual response variability, further optimization and new strategies are urgently needed.

Polysaccharides, as fundamental biomacromolecules within living organisms, play a crucial role in energy storage and structural support, and are also integral components of cell membranes. They play critical roles in biological processes and metabolic activities, such as cell adhesion, signal transduction, and immune responses (Fujita et al., 2011). Research and applications of polysaccharides as therapeutic agents have gained considerable attention since the 1960s, when lentinan was first reported to have anti-tumor effects (Chihara et al., 1969). Over the past 2 decades, 54 compounds containing oligosaccharides or polysaccharides as their primary structural units have received approval as drugs or diagnostic reagents globally (Cao et al., 2022). Polysaccharides from traditional Chinese medicine have unique pharmacological properties in immune regulation, anti-tumor, anti-diabetes, antioxidation, antiviral, cardiovascular protection, and neuroprotection. The primary sources of bioactive polysaccharides are derived from *Ganoderma lucidum*, *ginseng*, *Astragalus*, *lycium barbarum*, and *Angelica* (Sohretoglu et al., 2019; Li et al., 2019a; Jin et al., 2014a; Nai et al., 2021a).

*Astragalus membranaceus* (Fisch.) Bunge matched with *Angelica sinensis* (Oliv.) Diels, constitutes the traditional Qi-Gui herb pair, which was historically used for treating menstrual anemia and microvascular ischemic diseases (Pu et al., 2016). Contemporary pharmacological researches have demonstrated that this herbal combination exerts beneficial effects on hematopoiesis, immune modulation, cardiovascular protection, and anti-tumor activity (Feng et al., 2021). Polysaccharide is one of the main active ingredients shared by Astragalus and Angelica, which were found with significant effects of ameliorating anemia, combating aging, mitigating fibrosis, and anti-tumor. Polysaccharides from Astragalus and Angelica exhibit significant inhibitory effects on various tumors (Nai et al., 2021a; Shen et al., 2024; Li et al., 2020a). Astragalus polysaccharides demonstrated substantial impacts on various types of cancer, including lung, breast, and melanoma (Zhou et al., 2018a; Li et al., 2019b; Hwang et al., 2021). It executes an anti-tumor function by directly suppressing the proliferation of tumor cells, instigating apoptosis and cycle arrest, stimulating the immune system, and modulating the inflammatory microenvironment (Li et al., 2020a; Yu et al., 2019). Angelica polysaccharides demonstrated significant efficacy against leukemia, melanoma, colon, and liver cancer. Its anti-carcinogenic properties demonstrated by inhibiting tumor cell proliferation, inducing apoptosis, enhancing immune response, regulating autophagy, and preventing the tumor cells invasion and migration (Liu et al., 2020; Zhang et al., 2021). Compared with non-polysaccharide components, polysaccharide in Qi-Gui herb pair have stronger blood-replenishing effect, which can significantly promote bone marrow hematopoiesis, accelerate the differentiation and maturation of blood cells, and increase the number of peripheral blood red blood cells in mice with blood deficiency (Wang et al., 2021a). Furthermore, Qi-Gui polysaccharides have been shown to postpone cellular senescence, enhance immunological function, and mitigate oxidative harm to renal tissue in aged mice. However, little research has been reported about the homogeneous polysaccharide from Qi-Gui herb pair. AAPS-2A is the only reported homogeneous polysaccharide from Qi-Gui herb pair at present, which is composed of Rha, Gal, Ara and Glc in a molar ratio of 1:2.1:3.2:6.2, and exhibited strong antioxidant capacity *in vitro* (Pu et al., 2016). Since its identification, no additional homogeneous polysaccharides from the Qi-Gui herb pair have been discovered, nor has there been any investigation into their potential anti-tumor activity or underlying mechanisms. This lack of research highlights a substantial gap in the current understanding of these compounds.

This study isolated a homogeneous polysaccharide (AAPS-1a) from Qi-Gui herb pair, which was found composed of 1,6-α-D-Glc*p* (81.7%), T-α-D-Glc*p* (5.9%), 1,3-α-D-Gal*p* (3.9%), 1,4-α-D-Man*p* (3.6%), 1,4-α-D-Gal*p* (2.1%), and 1,4-α-D-Glc*p* (2.8%). Furthermore, AAPS-1a displays significant *in vitro* anti-tumor activity by inhibiting the proliferation of HCT116 cells, inducing G2 cycle arrest, and promoting apoptosis. These results suggest that AAPS-1a is a promising candidate for the treatment of colon cancer, which provide a theoretical basis for further research on the anti-colon cancer activity *in vivo* and structure-activity relationship.

## 2 Materials and methods

### 2.1 Materials and reagents

The fresh roots of *A. membranaceus* (Fisch.) Bunge and *A. sinensis* (Oliv.) Diels were obtained from Minxian County, Gansu Province, and identified by the experts at Shaanxi University of Chinese Medicine. DEAE Sephadex A-25 and Sephadex G-100 were obtained from GE Healthcare Ltd. (Chicago, USA). Standard monosaccharides including rhamnose (Rha), mannose (Man), galactose (Gal), galacturonic acid (GalA), glucuronic acid (GlcA), arabinose (Ara), glucose (Glc), and fucose (Fuc) were procured from Sigma-Aldrich, (St. Louis, MO, United States). T-series dextrans (T-5, T-12, T-50, T-150, T-410), penicillin and streptomycin were obtained from Sigma-Aldrich (St. Louis, MO, United States). RPMI 1640 and fetal bovine serum (FBS) were purchased from Gibco Life Technologies Co. (Grand Island, NY, United States). The Cell Counting Kit 8 (CCK-8) was obtained from the Beyotime Institute of Biotechnology. (Shanghai, China). Annexin V-FITC/PI kits were obtained from 4A Biotech Co., Ltd. (Beijing, China). All chemicals and reagents used in this study were of analytical grade.

### 2.2 Extraction and purification of AAPS-1a

The dried *A. membranaceus* (Fisch.) Bunge (5.0 kg) and *A. sinensis* (Oliv.) Diels (1.0 kg) were sliced, mixed, and refluxed with 12 L of ethanol (95%) at 25°C for 2 h, three times. Then the mixture was decocted twice with 0.1 mol/L NaOH at 80°C for 1 h. The extract was collected, filtered, and precipitated using 95% alcohol. Following the collection of the precipitate, it was re-dissolved in distilled water. The sevag reagent (n-butanol: chloroform = 1 : 4, v/v) was subsequently introduced into the solution, and the mixture underwent a freeze-thaw process for twenty cycles to facilitate the removal of protein. After dialysis, a 10% solution of H_2_O_2_ was added to the mixture to eliminate the pigments. The mixture was then dialyzed, and lyophilized to obtain the crude Qi-Gui polysaccharides (AAPS). AAPS was fractionated using a DEAE Sephadex A-25 column (120 cm × 5 cm) with distilled water as the eluent, and yield a fraction designated as AAPS-1. AAPS-1 was further fractionated by size-exclusion chromatography on a Sephadex G-100 column (120 cm × 5 cm), eluted with 0.1 M NaCl. The eluent was collected 5 mL per tube, the carbohydrate content in each tube was determined at 490 nm by phenol-sulfuric acid method. AAPS-1 was separated into two sub-fractions (AAPS-1a and AAPS-1b). Then AAPS-1a was dialyzed, lyophilized, and further purified using a Sephadex G-100 column eluted with distilled water, and resulting in the purified sub-fraction AAPS-1a.

### 2.3 Determination of carbohydrate and uronic acid

The contents of carbohydrate in AAPS-1a were measured by PhOH-H_2_SO_4_ method (Duan et al., 2022). The uronic acid content in AAPS-1a were measured via the vitriol-carbazole method (Filisetti-Cozzi and Carpita, 1991).

### 2.4 Fourier transform infrared spectrum analysis

AAPS-1a (2 mg) was vacuum-dried at 35°C–44°C, mixed with dried potassium bromide, and pressed into a pellet. Its FT-IR spectrum was captured using a Tensor 27 FT-IR spectrometer (Bruker, MA, United States) with 64 scans at a 4 cm^−1^ resolution in the mid-infrared range (4,000–400 cm^−1^).

### 2.5 Molecular weight and homogeneity of AAPS-1a

High-performance size exclusion chromatography (HPSEC) was employed to determine the molecular weights and homogeneity of AAPS-1a (Wang et al., 2021b). In summary, AAPS-1a and standard dextrans were dissolved in distilled water and analyzed using a Waters Alliance 2,695 system equipped with a TSK-GEL G3000 PWXL column (TOSOH, 7.8 mm × 30.0 cm) and a Waters Alliance 2414 RI detector. 0.06 M Na_2_SO_4_ solution was used as the eluent at a flow rate of 0.6 mL/min. A standard curve was constructed by plotting the logarithm of molecular weights of standard dextrans against their Kav, where Kav = (*V*_e_ - *V*_o_)/(*V*_t_ - *V*_o_): *V*_e_, elution volume; *V*_o_, void volume; *V*_t_, total bed volume. *V*_t_ is the total volume determined by glucose, and *V*_o_ is the void volume determined with blue dextran 2000. The molecular weight of AAPS-1a was determined by applying its retention time to the standard curve equation.

### 2.6 Monosaccharide composition of AAPS-1a

The monosaccharide composition of AAPS-1a was determined by derivatizing it with 1-phenyl-3-methyl-5-pyrazolinone (PMP) and analyzing it using high-performance liquid chromatography (HPLC) (Liu et al., 2022). AAPS-1a was hydrolyzed with TFA (2 M) at 100°C for 8 h. The resulting hydrolysate was treated with 0.3 M NaOH and 0.5 M PMP for 0.5 h, after adjusting the pH to 7.0. The derivatives were extracted using CH_3_Cl and collected for HPLC analysis. The standard monosaccharides underwent the same modification procedures as AAPS-1a. The derivatives were analyzed using a DIONEX UltiMate system with an AccliamTM 120 C18 column and an Ultimate 3,000 Photodiode Array detector. The mobile phase was composed of 100 mM ammonium acetate, tetrahydrofuran, and acetonitrile in an 81:2:17 ratio.

### 2.7 Methylation analysis

The linkage types of sugar residue in AAPS-1a were analyzed by GC-MS following methylation (Needs and Selvendran, 1993). AAPS-1a was combined with anhydrous DMSO and stirred for 12 h. Subsequently, a DMSO/NaOH solution was added and mixed for 3 h. The mixture was then treated with 1 mL of methyl iodide, stirred for 7 min in an ice-cold water bath, and maintained at 37°C for 12 h. This process was repeated three times, and the final solution was extracted using chloroform. Following this, the extract was hydrolyzed by HCOOH and TFA, reduced by sodium borodeuteride, and acetylated by acetic anhydride in pyridine. The acetylated product was finally extracted with chloroform, and analyzed with a GC-MS QP2010 Ultra system (Shimadzu, Japan) equipped with a DB-1 capillary column (30 m × 0.25 mm × 0.15 µm). Helium was used as the carrier gas at a flow rate of 1 mL/min. The temperature program was: 45°C for 5 min, ramping to 100°C at 10°C/min, and holding for 5 min, increasing to 170°C at 0.5°C/min and holding for 1 min, then ramping to 280°C at 15°C/min, and holding for 5 min. Specimens were loaded in splitless mode with the injector and detector set at 220°C and 280°C, respectively. Methylated alditol acetates were identified by their retention times and fragment ions detected via Mass Spectrometry (MS), while Gas Chromatography (GC) peaks enabled the analysis of the relative content of each sugar residue.

### 2.8 Nuclear magnetic resonance spectroscopy

50 mg of AAPS-1a was dissolved in 1 mL D_2_O and lyophilized three times, then the samples were dissolved in 500 μL D_2_O. The ^1^H NMR, ^13^C NMR and 2D spectra, including ^1^H–^1^H COSY, HSQC, HMBC, NOESY, were detected at 500 MHz.

### 2.9 CCK-8 assay

HCT116, HepG2, and Caco2 cells were kindly provided by Xijing Hospital of Digestive Diseases of the Fourth Military Medical University, which were cultured in RPMI-1640 medium supplemented with 10% FBS and 1% penicillin and streptomycin in a humidified incubator with 5% CO_2_. Cells in the logarithmic growth phase were seeded in 96-well plates (1 × 10^3^/well) and treated with AAPS-1a (0.03–100 mg/L) for 48 h, 6 wells were set for each group. Following this, CCK-8 reagent was added and incubated for 2 h, after which the optical density (OD) at 450 nm was measured. The inhibition rate was calculated using the formula: [(OD _control_–OD _sample_)/OD _control_] × 100%.

### 2.10 Flow cytometry

HCT116 cells (6 × 10^4^/well) were seeded in 6-well plates, treated with AAPS-1a for 48 h, and stained with propidium iodide (PI) following the manufacturer’s protocol of Cell Cycle Detection Kits manufacturer’s protocol. Four replicates were set in each group. The cells were analyzed using flow cytometry (FACS Calibbur, BD, New Jersey, US) and CELL QUEST PRO. The experiments were conducted in triplicate.

### 2.11 Statistical analysis

Data are presented as mean ± S.E.M. Bio-assay data were statistically analyzed using Student’s t-test or ANOVA with subsequent *post hoc* analysis. *P < 0.05 was considered significant, and **P < 0.01 was considered highly significant.

## 3 Results

### 3.1 Purification, homogeneity and molecular weight of AAPS-1a

**FIGURE 1:**
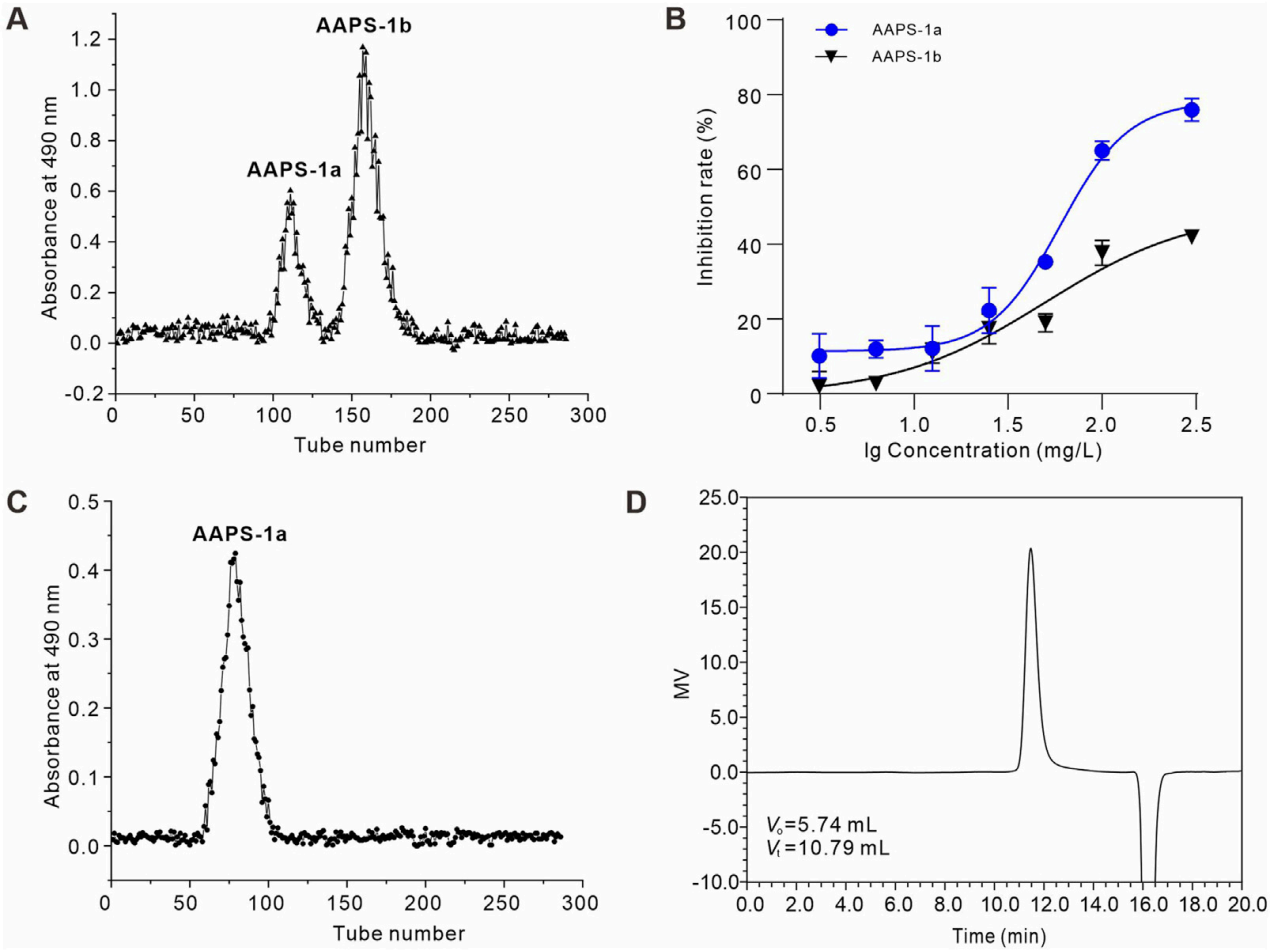
Isolation and purification of AAPS-1a from the Qi-Gui herb pair. **(A)** Elution profile of AAPS-1 on a Sephadex G-100 column. **(B)** Effects of AAPS-1a and AAPS-1b on HCT116 cells viability (n = 6). **(C)** Elution profile of AAPS-1a by Sephadex G-100 column. **(D)** HPSEC chromatogram of AAPS-1a on a G3000 PWXL column.

The total polysaccharide (AAPS) was extracted from the Qi-Gui herb pair and purified using DEAE Sephadex A-25 and Sephadex G-100 columns. AAPS was initially purified using DEAE Sephadex A-25 with distilled water elution, yielding two fractions: AAPS-1a and AAPS-1b (Figure 1A). The yields of AAPS-1a and AAPS-1b relative to AAPS were 13.1% and 25.5%, respectively. AAPS-1a exhibited greater inhibition of HCT116 cell proliferation (Figure 1B) and was subsequently selected for further purification using a Sephadex G-100 column (Figure 1C). The pure eluted fraction was collected and analyzed using HPSEC. The HPSEC profile of AAPS-1a displayed a singular, symmetrical and sharp peak, indicating its homogeneity as a polysaccharide (Figure 1D). The average molecular weight was estimated to be 17.5 kDa based on the calibration equation derived from the linear regression analysis of the calibration curve.

### 3.2 Identification and monosaccharide composition of AAPS-1a

**FIGURE 2:**
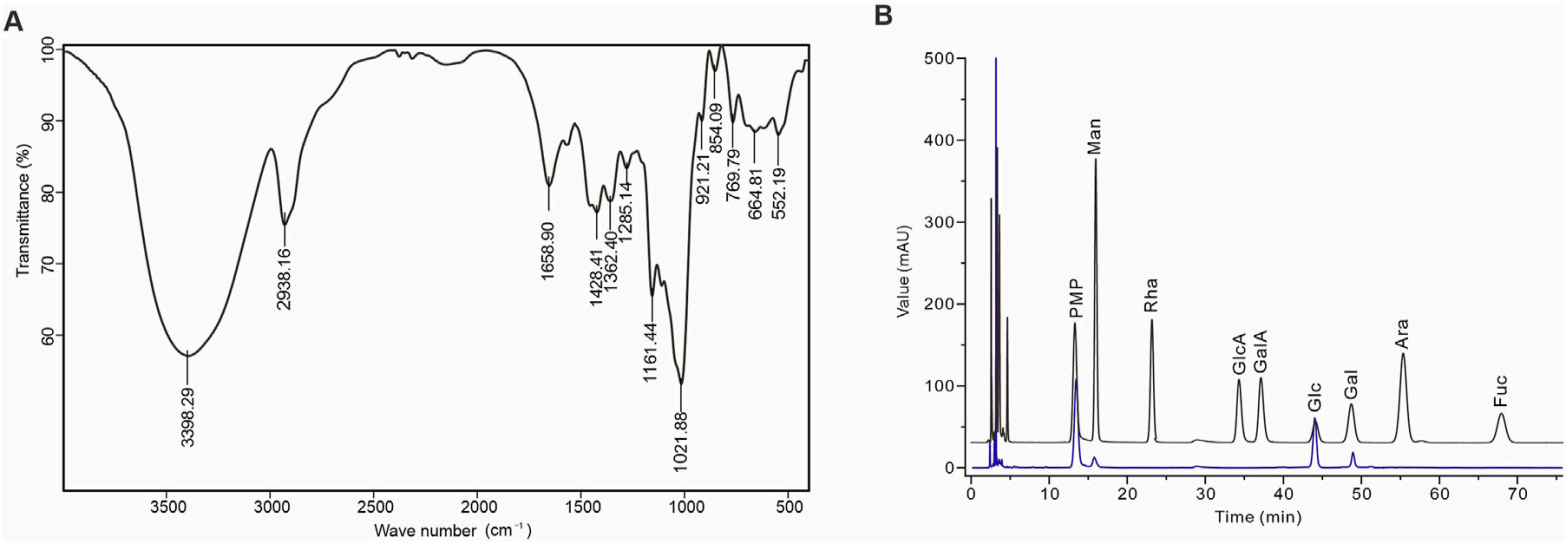
Identification and monosaccharide composition of AAPS-1a. **(A)** IR spectrum of AAPS-1a. **(B)** HPLC chromatogram of AAPS-1a.

AAPS-1a comprised 97.43% carbohydrates, with uronic acid and protein present in trace amounts (less than 2%). Moreover, the FT-IR spectrum of AAPS-1a displayed the typical polysaccharide characteristics (Meng et al., 2014). As shown in Figure 2A, the broad signal at 3,398.29 cm^−1^ is corresponds to the O-H stretching vibration. The signals at 2,938.16 and 1,428.41 cm^−1^ are due to the symmetric and asymmetric stretching vibrations of C-H bonds. The signal near 1,658.90 cm^−1^ is corresponding to the carbonyl stretching vibrations. The bands at 1,362.40, 1,285.40, and 1,021.88 cm^−1^ correspond to the C-O stretching vibrations of a pyran ring, with additional contributions from C-C-H and C-O-H deformations (Meng et al., 2014). The bands at 854.09 and 921.21 cm^−1^ correspond to the α-configuration and β-configuration in the sugar units (Kan et al., 2020). The signal at 1,161.44 cm^−1^ corresponds to the molecular bending vibration of the polysaccharide. HPLC further revealed that AAPS-1a was composed of Man, Glc and Gal in a molar ratio of 1.0:27.1:2.2 (Figure 2B).

### 3.3 GC-MS analysis of AAPS-1a

**FIGURE 3:**
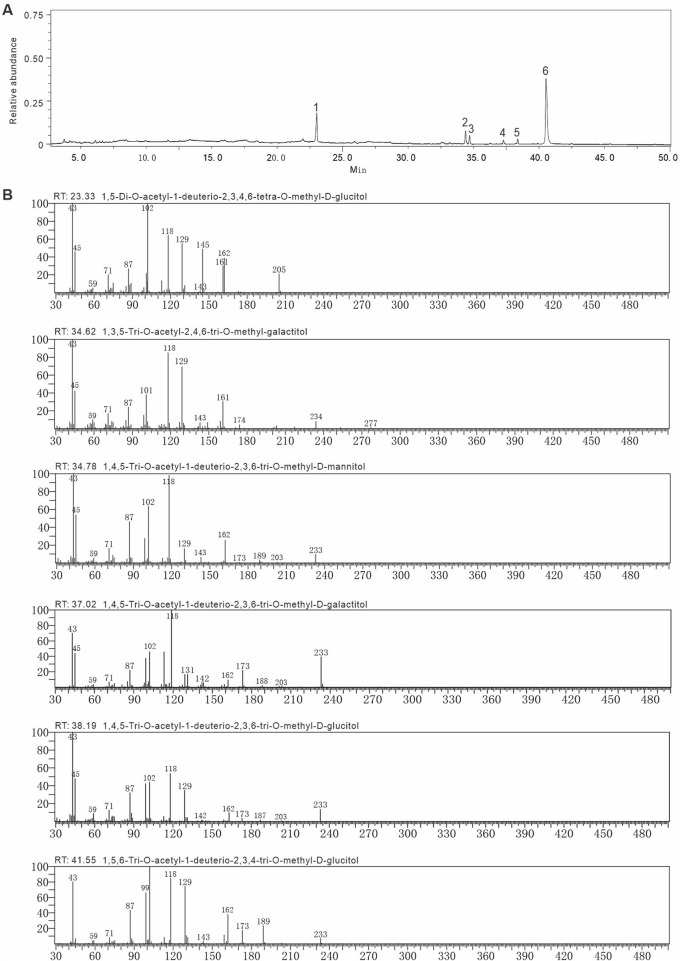
Methylation and GC-MS of AAPS-1a. **(A)** GC chromatogram of AAPS-1a. **(B)** The fragment ions of MS of peaks in methylated alditol acetate of AAPS-1a.

Methylation and GC-MS analysis identified the types and linkages of sugar residues in AAPS-1a. The GC chromatogram of AAPS-1a revealed six peaks with retention times at 23.33, 34.62, 34.79, 37.02, 38.19, and 41.55 min (Figure 3A). By comparing the relative retention times and MS fragment ions of each peak with literature data (Liu et al., 2018; Chen et al., 2018) and the PMAA database (https://glygen.ccrc.uga.edu/ccrc/specdb/ms/pmaa/pframe.html), the six peaks were identified as PMAA of 1,5-Di-O-acetyl-2,3,4,6-tetra-O-methyl-D-glucitol, 1,3,5-Tri-O-acetyl-2,4,6-tri-O-methyl-galactitol, 1,4,5-Tri-O-acetyl-1-deuterio-2,3,6-tri-O-methyl-D-mannitol, 1,4,5-Tri-O-acetyl-2,3,6-tri-O-methyl-galactitol, 1,4,5-Tri-O-acetyl-1-deuterio-2,3,6-tri-O-methyl-D-glucitol, and 1,5,6-Tri-O-acetyl-1-deuterio-2,3,4-tri-O-methyl-D-glucitol (Figure 3B). Finally, AAPS-1a was identified to be composed of T-Glc*p* (5.9%), 1,3-Gal*p* (3.9%), 1,4-Man*p* (3.6%), 1,4-Gal*p* (2.1%), 1,4-Glc*p* (2.8%), and 1,6-Glc*p* (81.7%) (Table 1).

**TABLE 1:** The methylation analysis of AAPS-1a using GC-MS.

Linkage type	Retention time	Molar ratio`	Partially methylated alditol acetates (PMAAs)	Mass fragments (m/z)
T-Glc*p*	23.33	2.3	1,5-Di-O-acetyl-2,3,4,6-tetra-O-methyl-D-glucitol	45, 118, 161, 162, 205
	(1.000)^a^	(5.9%)^b^		
1,3-Gal*p*	34.62	1.5	1,3,5-Tri-O-acetyl-2,4,6-tri-O-methyl-galactitol	45, 118, 161, 234, 277
	(1.484)	3.9%		
1,4-Man*p*	34.79	1.4	1,4,5-Tri-O-acetyl-1-deuterio-2,3,6-tri-O-methyl-D-mannitol	45, 118, 162, 233
	(1.491)	3.6%		
1,4-Gal*p*	37.02	0.8	1,4,5-Tri-O-acetyl-2,3,6-tri-O-methyl-galactitol	45, 118, 162, 233
	(1.587)	2.1%		
1,4-Glc*p*	38.19	1.1	1,4,5-Tri-O-acetyl-1-deuterio-2,3,6-tri-O-methyl-D-glucitol	45, 118, 162, 233
	(1.637)	2.8%		
1,6-Glc*p*	41.55	31.7	1,5,6-Tri-O-acetyl-1-deuterio-2,3,4-tri-O-methyl-D-glucitol	118, 162, 189, 233
	(1.781)	81.7%		

^a^ Values between parentheses indicated retention times relative to T-Glc*p*.

^b^ Percentage of residues in AAPS-1a.

### 3.4 1D and 2D NMR analysis of AAPS-1a

**FIGURE 4:**
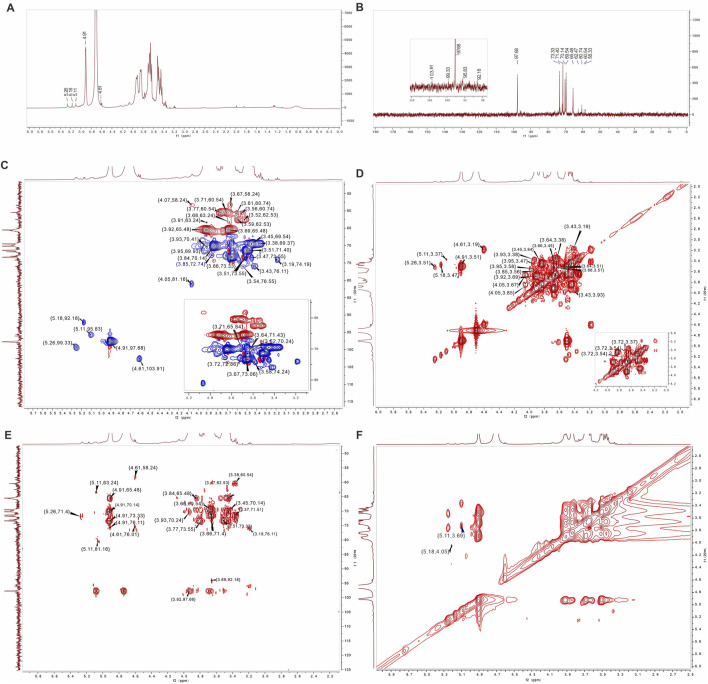
1D and 2D NMR spectra of AAPS-1a. **(A)** ^1^H NMR. **(B)** ^13^C NMR. **(C)** HSQC. **(D)** COSY. **(E)** HMBC. **(F)** NOESY.

In the ^1^H NMR spectrum of AAPS-1a, five anomeric proton signals at 4.61, 4.91, 5.11, 5.18, and 5.26 ppm were observed (Figure 4A). In the ^13^C NMR spectrum of AAPS-1a, five anomeric carbon signals at 92.18, 95.83, 97.68, 99.33, and 103.91 ppm were observed (Figure 4B). Combined with HSQC, five anomeric cross signals at *δ* 4.91/97.68, *δ* 4.61/103.91, *δ* 5.18/92.18, *δ* 5.26/99.33, and *δ* 5.11/95.83 were obtained (Figures 4C, D). The strong anomeric signal at *δ* 4.91/97.68 was attributed to residue “a”. Four signals at *δ* 4.91/3.51, *δ* 3.51/3.66, *δ* 3.66/3.45, and *δ* 3.45/3.84 in ^1^H,^1^H COSY indicated the correlation of H-1/H-2, H-2/H-3, H-3/H-4, and H-4/H-5 of residue “a” (Figure 4E), and two signals at *δ* 4.91/70.14, *δ* 4.91/65.48 in HMBC further helped to obtain the C-5 and C-6 (Figure 4F). Combined with HSQC and reported literature (Zhang et al., 2017), C-1∼C-6 of residue “a” were all assigned and residue “a” was identified to be 1,6-α-D-Glc*p*. Moreover, four cross signals at *δ* 3.51/3.64, *δ* 3.64/3.38, *δ* 3.38/3.93, *δ* 3.77/3.93 in COSY indicated the existence another Glc*p*, which was designated as residue “b”. Combined with five signals at *δ* 3.64/71.43, *δ* 3.38/69.37, *δ* 3.93/70.41, *δ* 3.71/60.54, *δ* 3.77/60.54 in HSQC and two signals at *δ* 3.77/73.55, *δ* 3.38/60.54 in HMBC, the H-3/C-3∼H-6/C-6 of residue “b” was obtained and residue “b” was identified to be T-α-D-Glc*p*.

The anomeric signal at *δ* 4.61/103.91 was designated as residue “c”. Three signals at *δ* 4.61/3.19, *δ* 3.19/3.43, and *δ* 3.43/3.91 in COSY indicated the H-1/H-2, H-2/H-3, and H-3/H-4 correlation. Based on three signals at *δ* 3.19/74.19, *δ* 3.43/76.11, and *δ* 3.91/70.61 in HSQC, the C-1, C-2, C-2, and C-4 of residue “c” were assigned. Combined with *δ* 3.19/76.11, *δ* 4.61/58.37 in HMBC, the C-3 and C-6 of residue “c” were assigned (Perepelov et al., 2013). The residue “c” was assigned to β-1,3-Gal*p* (Wei et al., 2018). Another three signals at *δ* 5.18/3.47, *δ* 3.47/3.95, and *δ* 3.95/3.58 confirmed the correlation of H-1∼H-4 of residue “d”. Based on *δ* 5.18/92.18, *δ* 3.47/73.55, *δ* 3.95/69.93 and *δ* 3.58/74.24, the C-1∼C-4 of residue “d” was obtained. A signal at 3.47/62.53 in HMBC further confirmed the H-2/C-6 correlation of residue “d”, which was identified to be 1,4-α-D-Gal*p* (He et al., 2020; Costa et al., 2019). A signal at *δ* 5.26/3.51 indicated the H-1/H-2 correlation of residue “e”. Based on *δ* 4.05/3.67, *δ* 4.05/3.85, *δ* 3.56/3.85, *δ* 3.61/3.85 in COSY, and *δ* 3.67/73.06, *δ* 4.05/81.16, *δ* 3.85/72.74, *δ* 3.56/60.74, and *δ* 3.61/60.74, the H-3/C-3∼H-6/C-6 of residue “e” were obtained. Residue “e” was identified to be 1,4-α-D-Man*p* (Cai et al., 2018; Meng et al., 2011). In addition, three signals at *δ* 5.11/3.37, *δ* 3.37/3.72, and *δ* 3.72/3.54 indicated the H-1/H-2, H-2/H-3, and H-3/H-4 correlation of residue “f” respectively. Combined with *δ* 5.11/95.83, *δ* 3.37/80.49, *δ* 3.72/72.86, and *δ* 3.54/76.01, and *δ* 5.11/63.63 in HMBC, C-1∼C-6 of residue “f” were all assigned, and residue “f” was identified to be α-1,4-Glc*p*. Finally, the he C/H chemical shifts of six residues were assigned and summarized in Table 2.

**TABLE 2:** ^1^H and ^13^C NMR spectral assignments for AAPS-1a.

Glycosyl residues		Chemical shifts, *δ* (ppm)					
		1	2	3	4	5	6
α-1,6-Glc*p* (a)	H	4.91	3.51	3.66	3.45	3.84	3.69/3.92
	C	97.68	71.4	73.33	69.54	70.14	65.48
α-T-Glc*p* (b)	H	4.91	3.51	3.64	3.38	3.93	3.71/3.77
	C	97.68	73.55	71.43	69.37	70.41	60.54
β-1,3-Gal*p* (c)	H	4.61	3.19	3.43	3.93	3.62	3.67/4.07
	C	103.91	74.19	76.11	70.41	70.24	58.24
α-1,4-Gal*p* (d)	H	5.18	3.47	3.95	3.58		3.52/3.59
	C	92.18	73.55	69.93	74.24		62.53
α-1,4-Man*p* (e)	H	5.26	3.51	3.67	4.05	3.85	3.56/3.61
	C	99.33	71.4	73.06	81.16	72.74	60.74
α-1,4-Glc*p* (f)	H	5.11	3.37	3.72	3.54	3.71	3.68/3.91
	C	95.83	69.37	72.86	76.01	69.84	63.24

Based on the HMBC and NOESY (Figures 4E, F), the linkage sequence of residues in AAPS-1a were further obtained., The signal at *δ* 5.18/4.05 in NOESY indicated the linkage between H-1 of α-1,4-Gal*p* and H-4 of α-α-1,4-Man*p*. The signal at *δ* 5.26/76.01 in HMBC indicated the linkage between H-1 of α-1,4-Man*p* and C-4 of α-1,4-Glc*p*. The signal at *δ* 5.11/3.69 in NOESY indicated the linkage between H-1 of α-1,4-Glc*p* and H-6 of α-1,6-Glc*p*. Another two signals at *δ* 4.91/65.48 and *δ* 3.92/97.68 indicated the correlation of H-1 and C-6 of α-1,6-Glc*p*. Moreover, a signal at 4.91/76.11 indicated the linkage between H-1 of α-1,6-Glc*p* and C-3 of α-1,3-Gal*p*.

### 3.5 The effect of AAPS-1a on tumor and normal cells proliferation

**FIGURE 5:**
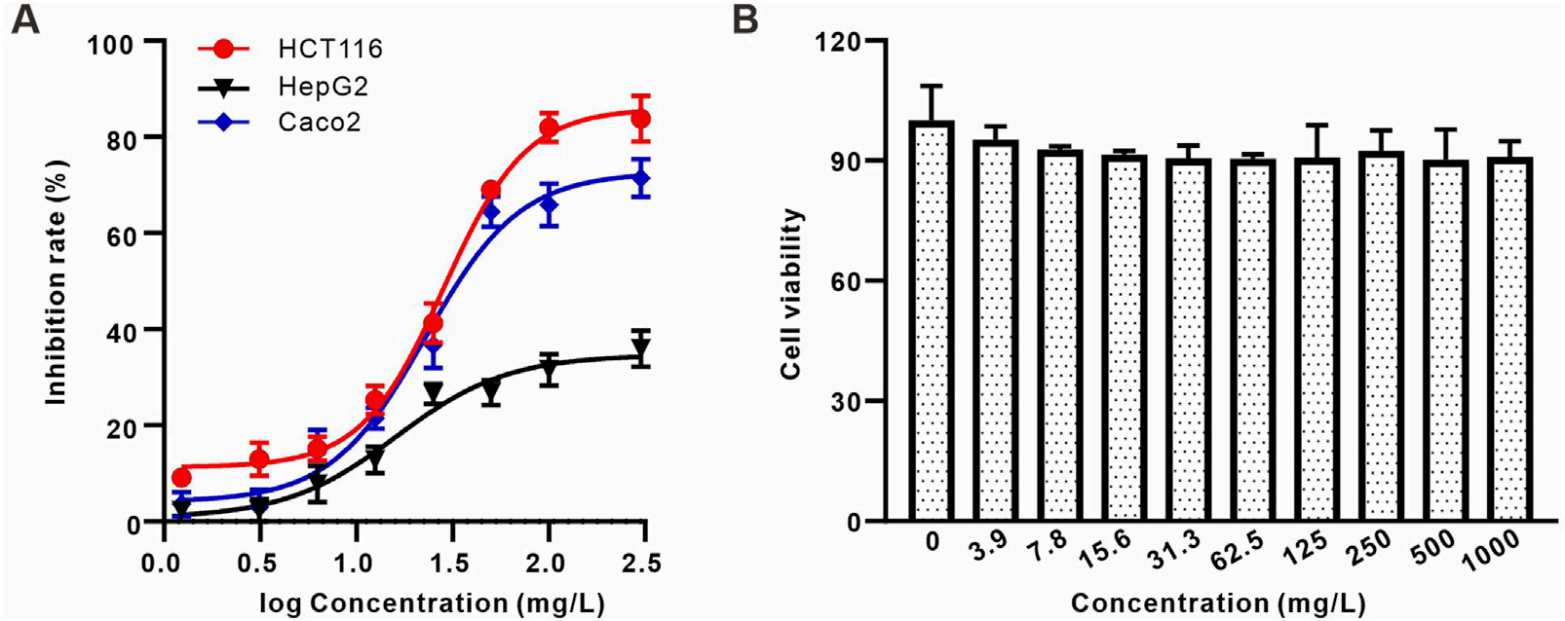
The effect of AAPS-1a on the proliferation of three tumor cell lines and normal cells **(A)** The effect of AAPS-1a on the proliferation of HCT116, HepG2 and Caco2 cells. **(B)** The effect of AAPS-1a on the proliferation of HIEC cells.

Polysaccharides derived from *Astragali* Radix and *Angelica Sinensis* Radix exhibit notable inhibitory effects on a variety of tumors, with a pronounced impact on colon and liver cancers (Jin et al., 2014b; Nai et al., 2021b). Therefore, to assess the anti-tumor efficacy of AAPS-1a, its influence on the proliferation of human colon cancer cell lines HCT116 and Caco2, in addition to the human hepatocellular carcinoma cell line HepG2, was systematically evaluated. AAPS-1a inhibited the proliferation of all three tumor cell lines in a concentration-dependent manner, achieving maximum inhibition rates of 81.97%, 65.85%, and 31.5%, respectively (Figure 5A). Furthermore, AAPS-1a demonstrated a significant inhibitory effect on the two human colon cancer cell HCT116 and Caco2, with an IC_50_ of 48.22 ± 3.4 μg/mL and 76.31 ± 5.2 μg/mL respectively, indicating its potential as a homogeneous polysaccharide with notable anti-colon cancer properties (Figure 5A). The HIEC cell line is a normal human intestinal epithelial crypt cell model (Gagné et al., 2020). Following the confirmed inhibitory effect of AAPS-1a on colon cancer cells proliferation, its impact on the normal proliferative HIEC cells was subsequently assessed. AAPS-1a showed no significant inhibition HIEC cells proliferation at concentrations ranging from 0.03 to 100 mg/L, indicating that it had no obvious cytotoxicity to normal colon cells (Figure 5B). Taken together, AAPS-1a has a strong cell proliferation inhibitory effect against colon cancer cells but no significant cytotoxicity to normal colon cells, demonstrating its’ selective inhibition towards cancer cells over normal cells.

### 3.6 The effect of AAPS-1a on the cell-cycle and apoptosis of HCT116 cells

**FIGURE 6:**
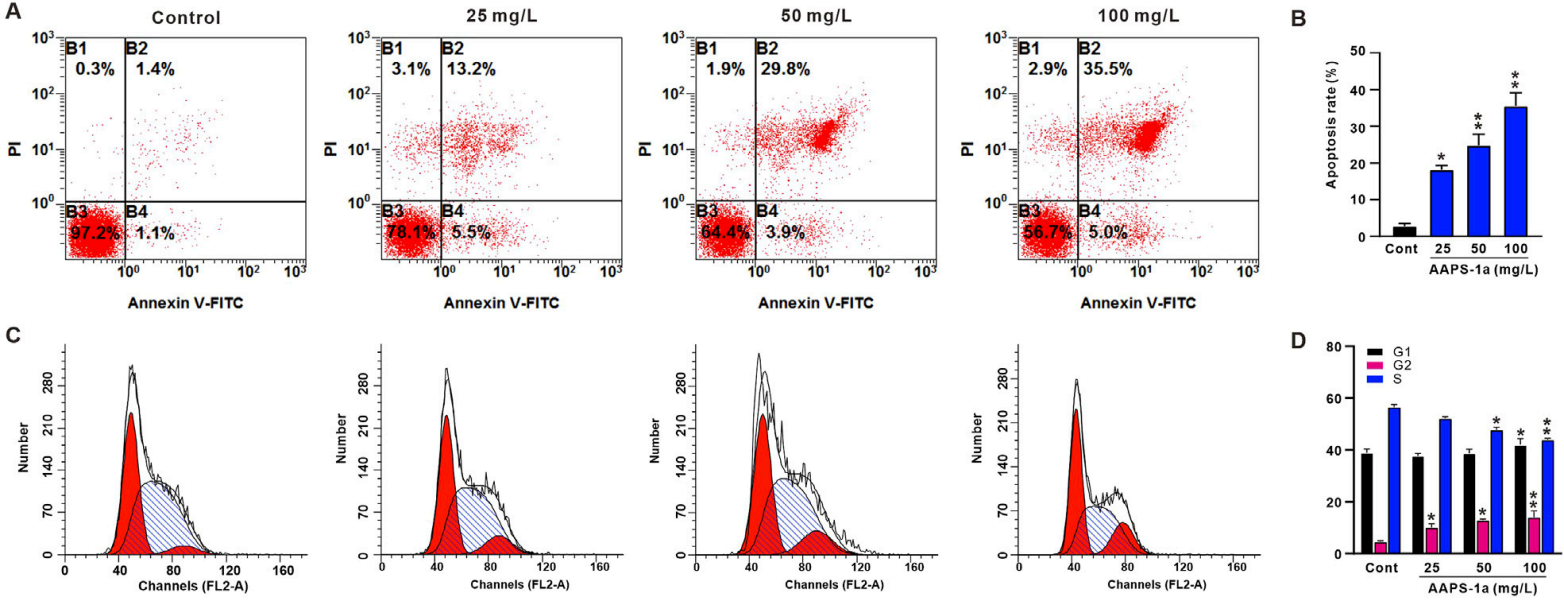
The effect of AAPS-1a on the cell-cycle and apoptosis of HCT116 cells. **(A)** The represented graphs of AAPS-1a on the apoptosis of HCT116 cells. **(B)** The statistical results of AAPS-1a on the HCT116 cells apoptosis (n = 4). **(C)** The represented graphs of AAPS-1a on the cells cycle of HCT116 cells. **(D)** The statistical results of AAPS-1a on the HCT116 cells cycle (n = 4). *P < 0.05 and **P < 0.01 compared with the control.

Given that AAPS-1a exhibited the most potent inhibitory effect on HCT116 cells, these cells were selected for subsequent assays. To examine if AAPS-1a inhibits HCT116 cell proliferation by affecting the cell cycle and inducing apoptosis, HCT116 cells were stained with PI and Annexin V-FITC, followed by flow cytometry analysis. Result of the cell cycle assay was displayed in Figures 6A, B. The S phase cell percentage serves as an indicator of cell proliferation. AAPS-1a reduced the S phase percentage from 56.5% ± 1.1% to 43.9% ± 0.6% and 36.0% ± 0.2% in a concentration-dependent manner. Moreover, the percentage of G2-phase cells were increased from 4.5% ± 0.4% to 14.1% ± 1.4%. AAPS-1a at high concentration (100 mg/L) increased the percentage of G1-phase cells from 38.9% ± 1.4% to 42.1% ± 2.3%, while low and moderate concentrations had no significant effect. The apoptosis assay result was displayed in Figures 6C, D. AAPS-1a demonstrated the markable apoptosis-inducing effect on HCT116 cells. Following AAPS-1a treatment, the apoptosis rate of HCT116 increased dose-dependently from 2.8% ± 0.7% to 35.5% ± 3.1%. These findings indicate that AAPS-1a inhibits HCT116 proliferation by inducing G2 cycle arrest and apoptosis.

## 4 Discussion

The compatibility of Astragalus and Angelica represents a classic herb pair for “Qi invigoration and blood supplementation” in TCM, which has been utilized for thousands of years (Barbosa and Carvalho Junior, 2020). Polysaccharides are among the primary active constituents in the Qi-Gui herb pair, which exhibits various effects, including hematopoietic promotion, immune regulation, and anti-tumor activity. However, the structural basis and mechanisms underlying the anti-tumor properties of Qi-Gui polysaccharides remain inadequately understood, necessitating further investigation. Our preliminary evaluation of two polysaccharides from the Qi-Gui herb pair on HCT116 cells revealed that AAPS-1a significantly inhibits HCT116 cell proliferation. Based on these findings, this study demonstrated that AAPS-1a possesses anti-cancer activity against colon cancer *in vitro* by inducing apoptosis and cell cycle arrest.

In this study, polysaccharides were extracted from the Qi-Gui herb pair using the water extraction and alcohol precipitation method. As a traditional method, it is extensively employed for extracting polysaccharides from medicinal plants, effectively preserving their biological activity and ensuring reproducibility (Jiang et al., 2015; Yang et al., 2024a). Although alternative methods, such as ultrasound-assisted and microwave-assisted extractions, can enhance yields, they pose a risk of degrading polysaccharide structures and compromising their biological activity due to excessive heat or mechanical disruption. Additionally, these methods present challenges in temperature regulation, which can further contribute to polysaccharide degradation (Zhou et al., 2018b; Wu et al., 2020). While enzyme-assisted extraction is effective, it is more complex and costly, necessitating careful enzyme selection and optimization (Yu et al., 2020a). This approach also involves longer extraction times and potential stability issues. The use of combined extraction techniques, including ultrasound-assisted enzyme extraction and ultrasound-microwave hybrid extraction, has the potential to improve extraction efficiency. However, these methods may also modify the structure and biological activity of polysaccharides, rendering them less suitable for the objectives of this study (Yi et al., 2020a). Consequently, the traditional water extraction followed by alcohol precipitation is favored, as it ensures the preservation of polysaccharide quality, activity, and structural integrity while circumventing the complexities and potential limitations associated with alternative methods. Thus, we have opted for the conventional water extraction and alcohol precipitation approach.

Polysaccharides have different properties that determine their anti-tumor activity, such as molecular weights, sugar residues composition, and water solubility. Studies have shown a strong correlation between the molecular weight of polysaccharides and their antitumor efficacy (Jiang et al., 2015). Polysaccharides with molecular weights between 20 kDa and 1,000 kDa generally demonstrate enhanced anti-tumor activity (Yang et al., 2024a). Yang et al. isolated five polysaccharides from the *Lactarius hatsudake* Tanaka mushroom: LHP-1 (898 kDa), LHP-2 (677 kDa), LHP-3 (385 kDa), LHP-4 (20 kDa), and LHP-5 (4.9 kDa). They found that LHP-4 and LHP-5 significantly inhibited tumor cell proliferation (Zhang et al., 2020). Zhou et al. found that the partial hydrolysates of a galactomannan (GalM40, 14.7 kDa) possessed higher anti-tumor activity than the original polysaccharide (GalM, 142.0 kDa) (Kadam et al., 2013). AAPS-1a is a homogeneous polysaccharide with molecular weight of 17.5 kDa. The low molecular weight of AAPS-1a may increase active sites, enhancing its anti-tumor activity. β-1,3-linkages in polysaccharides have been found to be predominant in antitumor and immune-regulating activities (Yi et al., 2020b; Chen et al., 2020). APS-4II is a polysaccharide from *A. sinensis* (Oliv.) Diels, containing 9.1% β-1,3-Gal*p*, demonstrated significant anti-melanoma activity (Wang et al., 2021a). An arabinogalactan from Jasmine tea, containing β-1,3-Gal*p* branch-chains, exhibited significant immunomodulatory effects on RAW264.7 macrophages (Huang et al., 2023). Furthermore, α-1,4-Glc*p* plays a role in the antitumor activity of polysaccharides by enhancing immune responses (Li et al., 2020b). Polysaccharides derived from the *L. hatsudake* Tanaka mushroom, which contain 1,4-Glc*p*, demonstrate potential antitumor properties (Yang et al., 2024b). A homogeneous polysaccharide isolated from *A. membranaceus*, comprising T-Glc*p*, 1,4-Glc*p*, and 1,4,6-Glc*p*, has been reported to exhibit significant antitumor and immunomodulatory activities (Chen et al., 2023).

AAPS-1a was found composed of α-T-Glc*p* (5.9%), β-1,3-Gal*p* (3.9%), α-1,4-Man*p* (3.6%), α-1,4-Gal*p* (2.1%), α-1,4-Glc*p* (2.8%), and α-1,6-Glc*p* (81.7%). The existence of β-1,3-Gal*p* and α-1,4-Glc*p* may contribute to the anti-tumor effect of AAPS-1a. Polysaccharides with good water solubility may demonstrate enhanced antitumor activities (Chen et al., 2009). The good water solubility also improved the anti-tumor activities of AAPS-1a. Taken together, the appropriate molecular weight and the presence of β-1,3-Gal*p* and α-1,4-Glc*p,* and the good water solubility of AAPS-1a may contribute to its anti-tumor effect, warranting further confirmation.

Cancer, a major global health issue, is the leading cause of morbidity and mortality, accounting for over 8.2 million deaths in recent years. Malignant tumors significantly threaten human health. Chemotherapy remains one of the primary systemic therapeutic approaches, albeit with notable side effects. Recent evidence indicates that polysaccharides from Traditional Chinese Medicine (TCM) have gained significant attention for their anticancer properties. Apoptosis is a critical mechanism through which these polysaccharides exert their antitumor effects. A polysaccharide from *Polygonum tenuifolia* inhibits SPC-A-1 cell proliferation through the FAS/FAS-L pathway (Yu et al., 2020b). A polysaccharide from *Cordyceps sinensis* inhibits HCT116 cell proliferation by inducing apoptosis and blocking autophagy flux via the PI3K-AKT-mTOR and AMPK-mTOR-ULK1 pathways (Qi et al., 2020). Xue et al. reported that Angelica polysaccharide alleviated hypoxia-induced apoptosis and autophagy in rat neural stem cells by downregulating BNIP3 (Xue et al., 2019). Our previous research showed that Angelica polysaccharide APS-2I exhibits anti-leukemia properties by binding to Galectin-3 and inducing apoptosis in leukemia cells (Zhang et al., 2021). In addition to directly inhibiting proliferation and inducing apoptosis, polysaccharides exhibit antitumor effects through immune regulation, gut microbiota, and gut metabolism, which are crucial in the anticancer properties of TCM polysaccharides (Liu et al., 2019). Astragalus polysaccharide combined with 5-FU enhances the anti-tumor effect and mitigates 5-FU-induced immunosuppression (Zhou et al., 2018a). Angelica polysaccharide APS-4II effectively induces a protective immune response and exhibits anti-melanoma effects (Wang et al., 2021a). Despite that significant efforts have been dedicated to discovering anticancer polysaccharides and complexes for developing effective cancer treatments, the underlying mechanisms *in vivo* need in-deep investigations.

## 5 Conclusion

The Qi-Gui herb pair, comprising *A. membranaceus* (Fisch.) Bunge and *A. sinensis* (Oliv.) Diels, has been a cornerstone of traditional medicine for tonifying Qi and nourishing Blood. In this study, we isolated a novel polysaccharide, AAPS-1a, with a molecular weight of 17.5 kDa, composed of Man, Glc, and Gal in a molar ratio of 1.0:27.1:2.2. Further structural analysis through GC-MS and NMR revealed that AAPS-1a consists of T-α-D-Glc*p* (5.9%), 1,3-α-D-Gal*p* (3.9%), 1,4-α-D-Man*p* (3.6%), 1,4-α-D-Gal*p* (2.1%), 1,4-α-D-Glc*p* (2.8%), and 1,6-α-D-Glc*p* (81.7%). The polysaccharide exhibits significant anti-colon cancer activity by inhibiting the proliferation of HCT116 cells, inducing G2 cycle arrest, and promoting apoptosis. These findings suggest that AAPS-1a holds promise as a potential therapeutic agent for colon cancer. This study underscores the need for future *in vivo* investigations to validate the therapeutic efficacy of AAPS-1a and further explore its mechanisms of action.

## Data Availability

The original contributions presented in the study are included in the article/supplementary material, further inquiries can be directed to the corresponding author.
